# Genome Sequence of *Propionibacterium acidipropionici* ATCC 55737

**DOI:** 10.1128/genomeA.00248-16

**Published:** 2016-05-19

**Authors:** Carlos H. Luna-Flores, Lars K. Nielsen, Esteban Marcellin

**Affiliations:** Australian Institute for Bioengineering and Nanotechnology, University of Queensland, Brisbane, Australia

## Abstract

*Propionibacterium acidipropionici* produces propionic acid as its main fermentation product. Traditionally derived from fossil fuels, environmental and sustainable issues have revived the interest in producing propionic acid using biological resources. Here, we present the closed sequence of *Propionibacterium acidipropionici* ATCC 55737, an efficient propionic acid producer.

## GENOME ANNOUNCEMENT

Traditionally derived from fossil fuels, propionic acid (PA) is widely used as a preservative. Increasingly used in other industries, the PA market has grown 5.1% per annum to over 350,000 tonnes per year (1). Mounting environmental concerns have renewed interest in finding a sustainable alternative for its production, reviving interest in bacterial fermentation (2). *Propionibacterium* spp. are pleomorphic rod-shaped, Gram-positive bacteria that naturally produce PA as their main fermentation product through the Wood-Werkman cycle (2, 3). Recent economic and environmental assessments have called for PA volumetric productivities of 2 g/(liter·h), concentrations of 100 g/L, and yields of 0.6 g/g for an economically viable process (1, 4). Sequencing of new *Propionibacterium* spp. strains should contribute to achieving those targets. Previously, *P. acidipropionici* ATCC 4875 was sequenced (3). In this study, we sequenced *P. acidipropionici* ATCC 55737, selected from a collection of 17 strains (5).

Sequencing was performed using PacBio and Illumina technologies as follows. DNA was extracted using a PureLink genomic DNA minikit (Invitrogen cat. no. K1820-01) and quantified using Nanodrop 1000 (Thermo Scientific) and Qubit (Life Technologies cat. no. Q32850). The quality of the DNA was determined by running a 1% agarose gel with the DNA gel stain SYBR safe (Life Technologies cat. no. S33102). The gel was visualized in a ChemiDoc MP system (Bio-Rad). The Illumina platform and the PacBio platforms were used for sequencing. The Illumina sequencing was performed using TrueSeq Illumina 300 paired-end. The library was prepared using the Illumina TrueSeq DNA HT sample preparation kit (Illumina cat. no. FC-121-2003). Reads were assembled using SPAdes (6). Genome closure was achieved using the PacBio RS II platform for sequencing. The PacBio library preparation was performed using the protocol for 20 kb selected with the BluePippin system. The chemistry used for sequencing was the release P6-C4. The sample was loaded using magnetic beads. The genome assembly was performed with the SMRT portal. This portal was also used to determine methylation sites across the genome. The Rapid Annotations using Subsystems Technology (RAST) server was used to annotate the assembled genome (7). *P. acidipropionici* ATCC 55737 has a genome of 3.71 Mb, a GC content of 68.7%, 3,406 coding sequences, and 65 RNAs. *P. acidipropionici* ATCC 55737 contains one plasmid with a genome size of 2,362 bp and a GC content of 67.69%.

In order to determine genomic differences between *P. acidipropionici* ATCC 4875 (3) and *P. acidipropionici* ATCC 55737 (this study), we performed a systematic genomic comparison. The genome size of the former strain is 3.66 Mb, which is 1.3% smaller than the newly sequenced strain. BLAST gene-gene comparison (8) showed that *P. acidipropionici* ATCC 4875 has 345 unique genes and that *P. acidipropionici* ATCC 55737 has 423 unique genes (*E* value <0.0001). All the genes involved in PA production were conserved within 98% (*E* value <0.0001). Major changes between the two strains were observed for the subsystem “prophages.” *P. acidipropionici* ATCC 55737 presents 30 prophage-associated proteins with a total size of 33,000 bp, whereas *P. acidipropionici* ATCC 4875 has only 17 prophage-associated proteins with a total size of 20,143 bp.

### Nucleotide sequence accession numbers.

Genome information for the chromosome and the plasmid of *P. acidipropionici* ATCC 55737 was deposited in the GenBank database under the accession numbers CP014352 and CP014353, respectively.

## References

[1] Rodriguez BA, Stowers CC, Pham V, Cox BM 2014 The production of propionic acid, propanol and propylene via sugar fermentation: an industrial perspective on the progress, technical challenges and future outlook. Green Chem 16:1066–1076. doi:10.1039/C3GC42000K.

[2] Liu L, Zhu Y, Li J, Wang M, Lee P, Du G, Chen J 2012 Microbial production of propionic acid from propionibacteria: current state, challenges and perspectives. Crit Rev Biotechnol 32:374–381. doi:10.3109/07388551.2011.651428. 22299651

[3] Parizzi LP, Grassi MC, Llerena LA, Carazzolle MF, Queiroz VL, Lunardi I, Zeidler AF, Teixeira PJ, Mieczkowski P, Rincones J, Pereira GA 2012 The genome sequence of *Propionibacterium acidipropionici* provides insights into its biotechnological and industrial potential. BMC Genomics 13:562. doi:10.1186/1471-2164-13-562. 23083487 PMC3534718

[4] Tufvesson P, Ekman A, Sardari RRR, Engdahl K, Tufvesson L 2013 Economic and environmental assessment of propionic acid production by fermentation using different renewable raw materials. Bioresour Technol 149:556–564. doi:10.1016/j.biortech.2013.09.049. 24103218

[5] Stowers CC, Cox BM, Rodriguez BA 2014 Development of an industrializable fermentation process for propionic acid production. J Ind Microbiol Biotechnol 41:837–852. doi:10.1007/s10295-014-1423-6. 24627047

[6] Bankevich A, Nurk S, Antipov D, Gurevich AA, Dvorkin M, Kulikov AS, Lesin VM, Nikolenko SI, Pham S, Prjibelski AD, Pyshkin AV, Sirotkin AV, Vyahhi N, Tesler G, Alekseyev MA, Pevzner PA 2012 SPAdes: a new genome assembly algorithm and its applications to single-cell sequencing. J Comput Biol 19:455–477. doi:10.1089/cmb.2012.0021. 22506599 PMC3342519

[7] Aziz RK, Bartels D, Best AA, DeJongh M, Disz T, Edwards RA, Formsma K, Gerdes S, Glass EM, Kubal M, Meyer F, Olsen GJ, Olson R, Osterman AL, Overbeek RA, McNeil LK, Paarmann D, Paczian T, Parrello B, Pusch GD, Reich C, Stevens R, Vassieva O, Vonstein V, Wilke A, Zagnitko O 2008 The RAST server: Rapid Annotations using Subsystems Technology. BMC Genomics 9:75. doi:10.1186/1471-2164-9-75. 18261238 PMC2265698

[8] Tatusova TA, Madden TL 1999 BLAST 2 sequences, a new tool for comparing protein and nucleotide sequences. FEMS Microbiol Lett 174:247–250. doi:10.1111/j.1574-6968.1999.tb13575.x. 10339815

